# Dietary supplementation of coconut meat modulates growth performance, nutritional composition, and internal regulation in Chinese mitten crab (*Eriocheir sinensis*)

**DOI:** 10.3389/fnut.2026.1757972

**Published:** 2026-03-06

**Authors:** Liangwei Xiong, Gang Jiang, Jia Wei, Yuanfeng Xu, Wenrong Feng, Jianlin Li, Yongkai Tang

**Affiliations:** 1 Department of Modern Fisheries, Jiangsu Agri-Animal Husbandry Vocational College, Taizhou, China; 2 Key Laboratory of Freshwater Fisheries and Germplasm Resources Utilization, Ministry of Agriculture and Rural Affairs, Freshwater Fisheries Research Center, Chinese Academy of Fishery Sciences, Wuxi, China; 3 Wuxi Fisheries College, Nanjing Agricultural University, Wuxi, China

**Keywords:** Chinese mitten crab, *Eriocheir sinensis*, coconut meat, growth, muscle quality, transcriptome

## Abstract

**Background:**

The Chinese mitten crab (*Eriocheir sinensis*) is a commercially important aquatic species valued for its high nutritional quality and desirable taste.

**Methods:**

This study investigated the effects of dietary supplementation with 30% coconut meat (experimental), compared to a basal diet of fresh-frozen fish (control), on male and female crabs. Growth performance, amino acid and fatty acid profiles in muscle and gonadal tissues were analyzed, and transcriptomic sequencing was conducted to elucidate the underlying molecular mechanisms.

**Conclusion:**

Results revealed a significantly higher CF values in females fed the coconut-supplemented diet (*P* < 0.05). Lauric acid (C12:0) content in gonads was significantly increased in the experimental group of male and female crabs, and female gonads also exhibited elevated polyunsaturated fatty acid (PUFA) levels (*P* < 0.05). In male crabs, muscle levels of the flavor-related amino acids glutamate, glycine, and alanine were significantly higher in the experimental group (*P* < 0.05). Furthermore, dietary coconut supplementation significantly enhanced overall antioxidant capacity, as indicated by improved GPx, CAT, and T-AOC values (*P* < 0.05). Transcriptome analysis highlighted 71 and 4,380 DEGs in experimental male and female crabs, respectively, relative to control group. KEGG enrichment analysis revealed that these DEGs were primarily involved in metabolic and developmental pathways, such as carbohydrate, lipid, and amino acid metabolism, Cell growth and death and endocrine. A total of 39 key DEGs were identified as central to the physiological responses induced by coconut supplementation. These findings provided a scientific basis for the use of coconut meal as a functional feed ingredient for Chinese mitten crab, and contributed to foundational knowledge for constructing gene regulatory networks related to nutritional quality in this species.

## 1 Introduction

Coconut (*Cocos nucifera L*.), a widely cultivated tropical crop (1, 2), yield approximately 55 million tons annually and is valued for its nutritional richness (3, 4). Its kernel is rich in lipids, proteins, carbohydrates, and minerals, with notable antioxidant potential (5). The primary processing by-product, coconut meal, retains these nutritional components and has been incorporated into various food products (6, 7). In addition to its nutritional value, coconut exhibits various biological activities relevant to animal health. Studies have demonstrated its antioxidant (8) and anti-inflammatory activities (9), with coconut kernel extract showing wound-healing effects and hydrolyzed coconut meal alleviating intestinal inflammation in animal models (10). These functional attributes, combined with its nutritional profile, support the potential of coconut meal as a functional feed ingredient in aquaculture to enhance animal health and mitigate oxidative stress.

Over the past few decades, the aquaculture sector has experienced a steadily increasing demand for formulated feeds. In crustacean nutrition, particularly for high-value species like crabs and shrimp, research on sustainable and effective feed formulations is critical. The exploration of alternative protein sources is driven not only by fishmeal scarcity and cost, but also by the need to optimize growth efficiency and product quality. Among various options, plant-based ingredients have become a major focus. Fishmeal has been extensively utilized in aquafeeds owing to its high nutritional quality, excellent digestibility, and balanced amino acid composition. However, in light of rising fishmeal prices, research on alternative plant-based protein sources has become increasingly critical. However, common plant proteins (e.g., soybean meal, cottonseed meal) often face limitations in crustacean feeds due to antinutritional factors, imbalanced amino acid profiles, and palatability issues, which can constrain growth performance and feed utilization efficiency (11, 12). Therefore, identifying novel plant ingredients that can overcome these drawbacks is of high priority. Al-Thobaiti et al. (13) reported that up to 20% of fishmeal can be replaced by plant proteins without compromising fish growth or health. Coconut meal, a promising plant-derived protein ingredient, offers considerable potential for integrated utilization and value-added development. Its moderate protein content, favorable fiber structure, and unique fatty acid profile position it as a potential functional feed component that may address some shortcomings of conventional plant proteins. It has been successfully incorporated into feeds for poultry such as chickens (14) and ducks (15), as well as for farmed fish species including *Epinephelus coioides* (16) and *Oreochromis niloticus* (17), with positive outcomes. Therefore, the inclusion of coconut meal as a sustainable protein substitute in aquafeeds presents significant advantages for resource conservation and reduction in production costs.

The Chinese mitten crab, *Eriocheir sinensis* (Phylum: Crustacea, Order: Decapoda, Family: Varunidae, Genus: Eriocheir), is a cornerstone species in China aquaculture, renowned for its esteemed flavor and nutritional profile. It commands significant economic value, with a nation production yield of 888,629 tons in 2023 (18), driven by robust domestic and seasonal international market demand, particularly as a premium autumn delicacy. However, meeting this growing demand is constrained by key bottlenecks in its intensive cultivation. Persistent issues such as suboptimal growth efficiency and feed conversion rates elevate production costs and environmental impact. The crab feed industry currently faces challenges due to the scarcity of high-quality feed ingredients and rising associated costs. As a result, the sector is compelled to incorporate alternative protein sources, which often exhibit inferior efficacy compared to fishmeal. These substitutes also present palatability issues; for instance, replacing 75% of fishmeal with cottonseed protein concentrate significantly reduces the growth performance and feed conversion efficiency of Chinese mitten crab (19, 20). These challenges underscore the urgent need to develop and evaluate innovative, sustainable feed formulations that can enhance growth efficiency, improve final product quality, and support the industry's sustainable development. In this context, evaluating coconut meal-a novel plant ingredient with suggested functional benefits-in the formulated feed for Chinese mitten crab directly addresses the industry's quest for improved feed formulations. This study therefore focuses on adult Chinese mitten crabs during the fattening stage. It aims to comprehensively evaluate the effects of dietary coconut meal supplementation, with emphasis on growth performance, nutritional quality, and underlying physiological regulation. The objective is to assess the feasibility of coconut meal in Chinese mitten crab aquaculture, enhance breeding efficiency and promote healthier farming practices, thereby providing a scientific basis for informed feed ingredient selection.

## 2 Materials and methods

### 2.1 Ethical statement

The use of animals in this study was performed in accordance with the guidelines for the care and use of animals for scientific purposes set by the Animal Ethics Committee of the Freshwater Fisheries Research Center, Chinese Academy of Fishery Sciences (Wuxi, China) (LAECFFRC-2023-06-12).

### 2.2 Test crab and feeding protocol

The Chinese mitten crabs used in this study were sourced from Yangcheng Lake Base of the Freshwater Fisheries Research Center of the Chinese Academy of Fishery Sciences (Suzhou, China). The juveniles were maintained in an indoor glass tank (100 cm × 45 cm × 50 cm) fed with fresh-frozen fish for 7 days.

### 2.3 Experimental design

After acclimatizing to laboratory conditions, 180 healthy male and female crabs (initial weight: 130.10 ± 23.83 g) were averagely divided into 4 groups and cultured in the identically sized tanks, including the male experimental group (MT), male control group (MC), female experimental group (FT), and female control group (FC). Each group consisted of three replicates with 15 crabs per tank. The male and female crabs in the control groups were fed fresh-frozen fish, while the crabs in the experimental groups received a diet comprising 70% fresh-frozen fish and 30% coconut meat. The crabs were fed twice daily (6:00 am, 17:00 pm) with a daily ration amount of 3%−5% of body weight for 4 weeks. Three hours after feeding, the feces and residues was collected to maintain water cleanliness. The frozen fresh fish used in the experiment were small anchovies, while the coconut meat was sourced from Wenchang, Hainan. Prior to feeding, the coconut meat was collected, ground into particles of approximately 1 mm in size, and thoroughly mixed with the frozen fish. Throughout the feeding trial, continuous aeration was provided via a blower, and periodic water- exchange with filtered water were performed to maintain water quality parameters. The water quality conditions during the experiment mirrored those of the acclimatization period, including water temperature (26 °C ± 0.5 °C), pH (7.5–7.6), and dissolved oxygen (>5 mg/L).

### 2.4 Sample collection

At the end of the feeding trial, all treatments were fasted for 24 h prior to final sampling. For physiological and biochemical analyses, six crabs were randomly selected from each tank (15 crabs per tank), anesthetized using 80 mg/L isoeugenol (ScanAqua, Norway), and sampled. After measuring the body weight and body length of each crab, hemolymph was collected from the fourth basal leg and used for enzymatic activities assays. The muscle and gonad were then dissected separately and used for determining the proximate compositions.

For molecular and microbial analyses, a separate batch of six different crabs was randomly selected from the remaining individuals in each tank. Muscle and gut tissues were excised from these crabs. The muscle samples were used for transcriptome sequencing, gill samples were used for the community composition analysis of intestinal microbiota. All tested tissues were immediately immersed in liquid nitrogen until further analysis.

### 2.5 Assay of antioxidant enzymes activity

The serum was collected by centrifugation at 3,000 × g at 4 °C for 10 min from hemolymph samples. Serum was used to measure the activities of total antioxidant capacity (T-AOC), superoxide dismutase (SOD), catalase (CAT), and glutathione peroxidase (GPx). The methods were conducted following the manufacturer's instructions provided by the commercial kits (Jiancheng Biotech Co.).

### 2.6 Muscle and gonad nutritional component

#### 2.6.1 Determination of fatty acid composition

The fatty acid profiles in muscle and gonad were analyzed using an Agilent 7890B5977A (Agilent Technologies, Santa Clara, USA) Gas Chromatograph with mass spectrum (GC-MS) equipped with SP™-2560 Silica Capillary Column (100 m × 0.25 mm × 0.2 μm film thickness, Supelco, Darmstadt, Germany). Nitrogen was used as a carrier gas at a linear velocity of 10 ml/min, and the inlet temperature was set to 260 °C with a split ratio of 20:1. The oven program was executed as follows: an initial temperature of 40 °C lowed by a 20 °C per minute ramp until reaching 100 °C, which was then isothermally maintained for 15 min. Subsequently, a 20 °C per minute ramp was applied until 190 °C, held isothermally for 6 min. Finally, a 1 °C per minute ramp was executed until reaching 220 °C, maintaining this temperature for 7 min.

#### 2.6.2 Amino acid content determination

According to the national food safety standard GB5009.124-2016, “Determination of amino acids in food”, acid hydrolysis method was used to determine the amino acid content of muscle and gonad, except for tryptophan, which was destroyed by hydrolysis. The specific method was as follows: 100 mg sample was accurately weighed into a hydrolysis tube, and 8 mL hydrochloric acid solution was added. After thorough mixing, the tube was flushed with nitrogen, sealed, placed in an oven at 120 °C, and hydrolyzed for 22–24 h. The hydrolysate was transferred to a volumetric flask, neutralized with 4.8 ml sodium hydroxide (10 mol/L), and then distilled water was added to complete the volume to 25 ml. Following filtration and centrifugation, 400 ml supernatant was transferred to a sample vial for amino acid by high-performance liquid chromatography (HPLC) using an Agilent 1100 instrument (Agilent, Santa Clara, CA, USA). The amino acid content of the samples was determined based on the peak area in the chromatogram.

### 2.7 Transcriptomic analysis using RNA-sequencing

The RNA pools of muscle and gonad from crabs were extracted using TRIzol reagent (Invitrogen, USA). The Agilent 2100 bioanalyzer was employed to assess the integrity and quality of the total RNA. The quality of RNA samples was suitable for transcriptomic analysis (1.80 < OD 260/280 < 2.00; OD 260/230 ≥2.0; RIN ≥8.0). The poly(A) mRNA was enriched using Oligo (dT) beads (Invitrogen, USA) and then cDNA was synthesized. Suitable fragments were selected as templates for PCR amplification and the library was then obtained. The construction, quality control and sequencing of RNA library were completed by Novogene Biotechnology Co., Ltd (Tianjin, China) using Hiseq 2000 (Illumina) platform.

The differentially expressed genes (DEGs) were identified using DESeq2 software. An FDR < 0.05 was considered as the differentially expressed genes (DEGs), log 2 fold change ≥1 was up-regulated, and log 2 fold change ≤ −1 was down-regulated. Furthermore, the DEGs were used for functional enrichment analysis, such as the Kyoto Encyclopedia of Genes and Genomes (KEGG) pathway and gene ontology (GO) terms analysis to explore meaningful biological functions and key signaling pathways.

### 2.8 Quantitative real-time PCR (qRT-PCR) validation

The accuracy of the transcriptome sequencing was confirmed using qRT-PCR analysis. Six DEGs were selected using the same RNA samples for Illumina transcriptome profiling. cDNA was synthesized utilizing a commercially available kit (#RR047A, Takara). Following this, 2 μl of the synthesized cDNA was utilized for the qPCR analysis, employing a commercially available kit (#RR820A, Takara) in conjunction with specific primers. The primers were designed using the Primer 5 software, and β-actin was used as an internal control (Table 1). The relative mRNA levels of each target gene were quantified according to the 2^−Δ*ΔCt*^ method (21). Three replicates were performed for each sample.

**Table 1:** Primers used for the qRT-PCR analysis.

**Gene**	**Sequence (F: 5^′^-3^′^)**	**Sequence (R: 5^′^-3^′^)**
*Hpd*	CACATCGTCGGCAACCAG	GAGCGGAAGAGCGGAGTA
*TCHH*	CAACACTTATCTGGAGCAA	TGGCGGAGTCTTATGGT
*Papss1*	CACCCGAAAGGAAGCCAGAT	GAAGCAAGAACACGCCAAGC
*Sort*	ATCCTGGCGGTGTTGAGA	TTCCTGGTTTCTGCTTCTTG
*PNP*	CTGCCATGAACAACGCCTAT	CCATATTGCCCTGCAAGTAG
*Klkb1*	CTCGGAGCAACGGATTTA	CACTCGTCCACCACCCTA
*β-actin*	TGGGTATGGAATCCGTTGGC	AGACAGAACGTTGTTGGCGA

### 2.9 Calculations and statistical analysis

The gonadosomatic index (GSI), weight gain rate (WGR), and condition factor (CF) of the experimental crabs are calculated using the following formulas:


$$\begin{array}{l} \text{GSI}\left(\text{\%}\right)=\left(\text{Gonadal weight}/\text{final weight}\right)\;\times \;100\%\\ \text{WGR}\left(\text{\%}\right)=\left[\left(\text{final weight }-\text{initial weight}\right)/\text{initial weight}\right]\;\times \;100\\ \text{CF}\left(\text{\%}\right)=\text{final weight}/\text{body lengt}{\text{h}}^{3}\;\times \;100 \end{array}$$

The obtained data were expressed as mean ± S.E.M. The statistical analysis was performed using an analysis of variance (SPSS 22.0) after exploring the normality and homogeneity of data. The difference between the same-sex experimental and the control groups was assessed using an independent sample *t*-test. To account for multiple comparisons and control the family-wise error rate, the False Discovery Rate (FDR) correction was applied, with the threshold for statistical significance set at *P* < 0.05.

## 3 Results

### 3.1 Growth performance

There were differences in growth performance between the MC and MT groups, as well as between the FC and FT groups (Table 2); however, these differences were not statistically significant. Over the 30-day experimental period, crabs fed coconut meat in the experimental groups exhibited a higher WGR than those in the control group, while no significantly differences were obtained (*P* > 0.05). Although the GSI was slightly elevated in both female and male crabs from the experimental groups compared to the control, no significant differences were observed (*P* > 0.05). Similarly, CF did not differ significantly between male crabs in the MC and MT groups (*P* > 0.05). In contrast, female crabs supplemented with dietary coconut meat showed a significantly higher CF (61.26 ± 7.42) compared to the control group (54.69 ± 2.57) (*P* < 0.05).

**Table 2:** Effect of coconut meat on the growth performance of Chinese mitten crab.

**Index**	Male		Female	
	**MC**	**MT**	**FC**	**FT**
Initial weight (g)	151.61 ± 7.66	154.47 ± 8.73	108.85 ± 4.37	105.5 ± 3.77
Final weight (g)	165.40 ± 8.59	170.54 ± 13.68	119.74 ± 4.81	118.43 ± 2.49
WGR (%)	14.89 ± 6.75	18.96 ± 2.84	15.25 ± 7.63	15.25 ± 3.32
GSI (%)	0.28 ± 0.10	0.30 ± 0.08	5.24 ± 0.76	5.26 ± 0.42
CF (%)	69.59 ± 4.94	70.20 ± 9.53	54.69 ± 2.57	61.26 ± 7.42^*^

Data were expressed as mean ± S.E.M from triplicate groups (*n* = 5). Asterisk indicates *P* < 0.05. WGR, weight gain rate; GSI, gonadosomatic index; CF, condition factor.

### 3.2 Antioxidant enzyme activities assays

**Figure 1:**
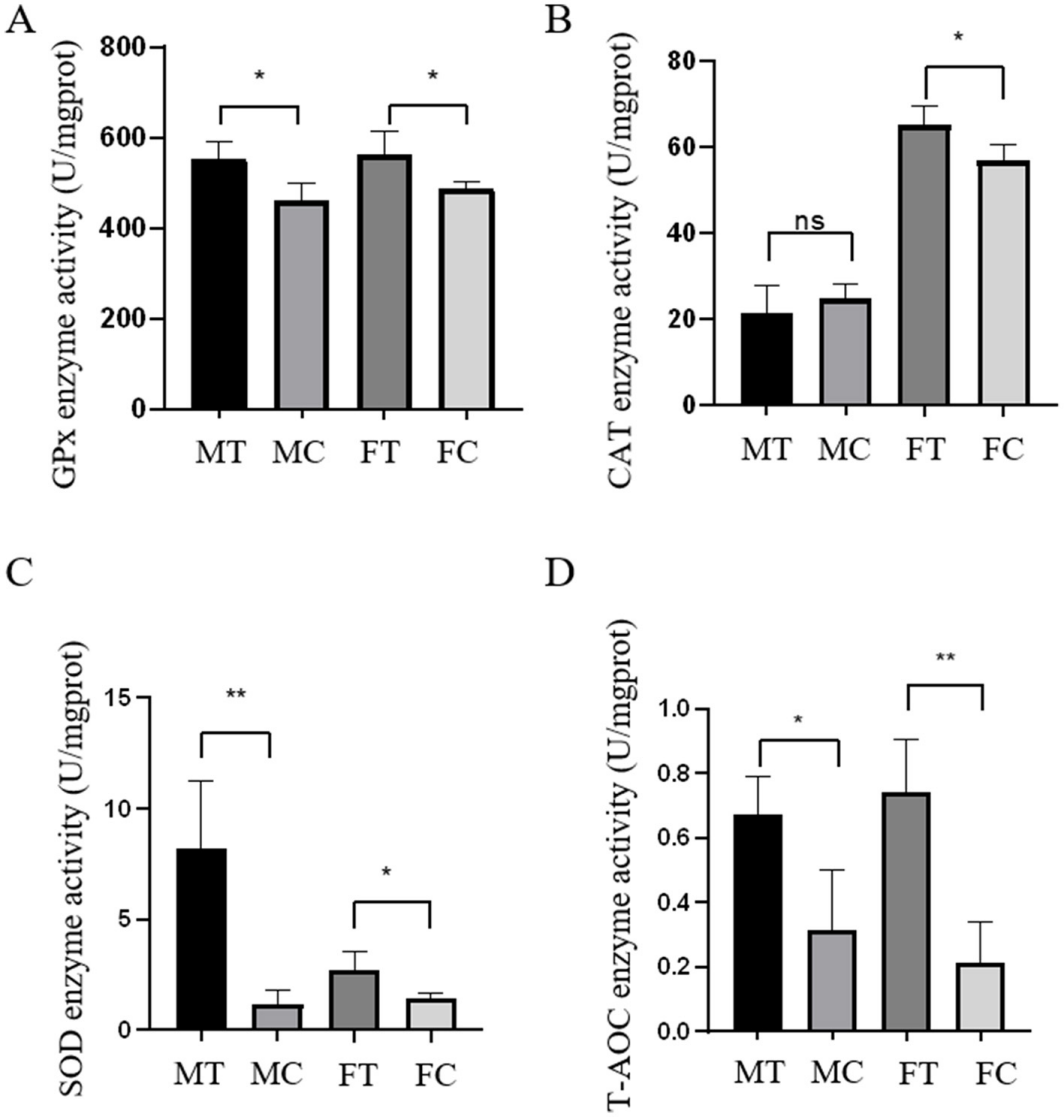
Effect of coconut meat on oxidase activity of Chinese mitten crab among control and experimental groups. **(A–D)** Represent the activities of GPx, CAT, SOD, and T-AOC, respectively. Mean ± SEM, *n* = 6). Asterisk indicates *P* < 0.05, double asterisk indicates *P* < 0.01.

The effects of dietary coconut meat supplementation on antioxidant enzyme activities in crabs are shown in Figure 1. In both male and female crabs, the experimental groups exhibited significantly higher activities of GSH-Px, SOD and T-AOC compared to the control groups (*P* < 0.05). For CAT activity in the serum, no significant difference was observed between the MT and the MC groups (*P* > 0.05). However, the FT group showed significantly higher CAT activity than the FC group (*P* < 0.05).

### 3.3 Fatty acid composition and content analysis

The types and contents of fatty acids in muscle were determined for the crab in the control and the experimental groups. As shown in Table 3, 25 fatty acids were detected in all groups, including 11 saturated fatty acids (SFA), 5 monounsaturated fatty acids (MUFA), and 9 polyunsaturated fatty acids (PUFA). This study found that in the fatty acid composition of the muscle and gonadal tissues of crab, C18:1n9c dominated, followed by C16:0 and C18:0. The experimental group was slightly higher than the control group, but there was no significant difference (*P* < 0.05).

**Table 3:** Fatty acid composition and content of muscle and gonadal tissue of Chinese mitten crab control and experimental groups.

**Fatty acid**	Muscles				Gonad			
	Male		Female		Male		Female	
	**MC**	**MT**	**FC**	**FT**	**MC**	**MT**	**FC**	**FT**
C12:0	0.00 ± 0.00	0.25 ± 0.23	0.00 ± 0.00	0.47 ± 0.05^*^	0.12 ± 0.03	1.24 ± 0.88^*^	0.05 ± 0.01	0.16 ± 0.05^*^
C14:0	0.45 ± 0.11	0.62 ± 0.22	0.33 ± 0.11	0.84 ± 0.1^*^	1.74 ± 0.96	2.34 ± 0.71	0.92 ± 0.08	1.25 ± 0.07
C15:0	0.3 ± 0.03	0.25 ± 0.07	0.24 ± 0.08	0.32 ± 0.13	0.57 ± 0.25	0.51 ± 0.04	0.42 ± 0.08	0.40 ± 0.04
C16:0	16.8 ± 1.85	17.8 ± 3.23	17.62 ± 4.15	20.49 ± 2.57	18.81 ± 3.77	18.92 ± 3.76	18.89 ± 0.68	18.19 ± 0.04
C16:1	2.94 ± 0.25	3.66 ± 1.49	2.97 ± 0.37	3.55 ± 0.48	5.17 ± 1.99	5.24 ± 2.99	9.75 ± 1.14	10.16 ± 1.45
C17:0	1.50 ± 0.14	1.19 ± 0.49	0.79 ± 0.27	0.93 ± 0.07	0.83 ± 0.36	0.86 ± 0.12	0.54 ± 0.13	0.55 ± 0.03
C18:0	18.38 ± 2.03	18.20 ± 1.71	15.53 ± 4.51	17.33 ± 1.34	10.2 ± 2.91	12.03 ± 2.62	6.70 ± 0.33	6.14 ± 0.33
C18:1n9c	29.3 ± 2.58	28.88 ± 0.85	30.22 ± 3.47	30.73 ± 0.89	25.47 ± 0.95	25.56 ± 1.13	32.53 ± 0.67	31.19 ± 1.14
C18:2n6c	4.94 ± 0.6	7.55 ± 3.83	6.58 ± 2.29	5.81 ± 1.26	4.24 ± 1.24	4.51 ± 0.49	9.73 ± 0.40	10.2 ± 0.44
C20:0	1.97 ± 0.12	1.72 ± 0.93	1.64 ± 0.43	2.06 ± 0.25	2.47 ± 0.99	3.08 ± 0.96	0.37 ± 0.05	0.40 ± 0.03
C18:3n3	0.26 ± 0.08	0.58 ± 0.54	0.41 ± 0.26	0.32 ± 0.11	0.32 ± 0.31	0.32 ± 0.05	1.34 ± 0.07	1.41 ± 0.09
C20:1	1.4 ± 0.13	1.27 ± 0.33	1.02 ± 0.22	1.37 ± 0.01	2.36 ± 0.25	2.13 ± 0.04;	0.54 ± 0.02	0.51 ± 0.07
C21:0	0.31 ± 0.02	0.27 ± 0.14	0.22 ± 0.05	0.28 ± 0.00	0.39 ± 0.16	0.46 ± 0.11	0.06 ± 0.02	0.11 ± 0.06
C20:2	1.72 ± 0.25	1.75 ± 0.29	1.37 ± 0.49	1.39 ± 0.5	2.31 ± 0.65	2.17 ± 0.28	0.97 ± 0.18	0.94 ± 0.10
C22:0	1.92 ± 0.03	1.63 ± 0.88	1.71 ± 0.29	2.14 ± 0.31	3.06 ± 1.20	4.10 ± 1.31	0.17 ± 0.02	0.20 ± 0.05
C20:3n6	0.07 ± 0.01	0.07 ± 0.02	0.1 ± 0.06	0.06 ± 0.01	0.19 ± 0.02	0.20 ± 0.06	0.11 ± 0.01	0.11 ± 0.01
C20:3n3	0.12 ± 0.04	0.18 ± 0.08	0.18 ± 0.14	0.12 ± 0.10	0.25 ± 0.10	0.18 ± 0.04	0.34 ± 0.02	0.36 ± 0.01
C22:1n9	4.19 ± 0.89	4.41 ± 1.57	3.92 ± 1.04	4.34 ± 0.84	3.00 ± 0.82	2.61 ± 0.54	1.27 ± 0.87	0.85 ± 0.18
C20:4n6	0.93 ± 0.21	1.89 ± 0.56^*^	1.82 ± 1.58	1.05 ± 0.48	6.68 ± 3.03	6.28 ± 0.26	1.98 ± 1.10	2.42 ± 0.18
C23:0	0.29 ± 0.02	0.26 ± 0.10	0.24 ± 0.05	0.30 ± 0.06	0.33 ± 0.11	0.43 ± 0.11	0.04 ± 0.01	0.08 ± 0.05
C22:2	0.09 ± 0.04	0.08 ± 0.01	0.09 ± 0.030	0.07 ± 0.02	0.17 ± 0.03	0.18 ± 0.06	0.04 ± 0.01	0.08 ± 0.06
C20:5n3	3.16 ± 2.95	5.75 ± 3.45	6.18 ± 5.39	4.16 ± 0.16	4.52 ± 2.89	4.23 ± 0.82	6.21 ± 0.81	6.99 ± 0.25
C24:0	0.49 ± 0.04	0.47 ± 0.08	0.47 ± 0.10	0.55 ± 0.02	0.65 ± 0.18	0.69 ± 0.04	0.13 ± 0.03	0.21 ± 0.00
C24:1	0.26 ± 0.03	0.3 ± 0.11	0.15 ± 0.06	0.22 ± 0.03	1.05 ± 0.41	1.29 ± 0.33	0.07 ± 0.02	0.10 ± 0.05
C22:6n3	3.39 ± 2.31	5.52 ± 4.39	6.20 ± 4.63	4.10 ± 1.23	5.01 ± 2.98	6.95 ± 1.07	6.84 ± 0.29	6.99 ± 0.01
∑SFA	42.40 ± 1.60	42.66 ± 3.43	38.8 ± 9.93	45.72 ± 4.81	39.17 ± 9.34	44.66 ± 3.86	28.29 ± 1.35	27.67 ± 0.54
∑MUFA	38.09 ± 2.24	38.53 ± 1.47	38.28 ± 5.16	40.21 ± 0.49	37.05 ± 1.25	36.82 ± 2.55	44.16 ± 1.74	42.82 ± 0.47
∑PUFA	15.17 ± 6.96	23.38 ± 12.64	22.92 ± 42.48	23.15 ± 10.55	23.78 ± 9.86	24.13 ± 2.09	27.55 ± 0.56	29.51 ± 1.01^*^

Data were expressed as mean ± S.E.M. Different superscript letters indicate significant differences (*P* < 0.05). Only the major fatty acids are shown in the table, and the detected fatty acids include C12:0, C14:0, C15:0, C16:0, C16:1, C17:0, C18:0, C18:1n9c, C18:2n6c, C18:3n3c, C20:1, C21:0, C20:2, C22:0, C20:3n6, C20:3n3, C22:1n9, C20:4n6, C23:0, C22:2, C20:5n3, C24:0, C24:1 and C22:6n-3. ∑MUFA, monounsaturated fatty acid; ∑PUFA, polyunsaturated fatty acid; ∑SFA, saturated fatty acid.

In male crabs, the experimental group exhibited lower concentrations of several fatty acids—including C15:0, C16:0, C18:0, C18:1n9c, C20:0, C21:0, C23:0, and C22:5—compared to the control group, while other fatty acids were relatively elevated. Notably, C20:4n6 (arachidonic acid, ARA) content was significantly higher in the experimental group (*P* < 0.05), and C12:0 (lauric acid) was detected exclusively in the experimental group, indicating a diet-specific metabolic shift.

In female muscle tissue, a similar modulatory effect was observed. The level of C14:0 levels were significantly higher in the experimental group (*P* < 0.05). Although the experimental group showed marginally higher total contents of saturated (SFA), monounsaturated (MUFA), and polyunsaturated (PUFA) fatty acids contents compared to the control, these differences were not statistically significant (*P* > 0.05).

Furthermore, dietary coconut meat supplementation significantly increased C12:0 levels in the gonadal tissue of Chinese mitten crabs (*P* < 0.05). In ovarian tissue, ∑PUFA content was significantly elevated compared to the control (*P* < 0.05), while other fatty acids remained unchanged (*P* > 0.05).

### 3.4 Amino acid composition and content analysis

In this study, either the muscle or gonad tissues, 17 amino acids were all detected in both control and experimental groups, consisting of 7 essential, and 10 non-essential amino acids (Table 4).

**Table 4:** Amino acid composition and content of muscle and gonadal tissue of Chinese mitten crab among control and experimental groups.

**Amino acid**	Muscles				Gonad			
	Male		Female		Male		Female	
	**MC**	**MT**	**FC**	**FT**	**MC**	**MT**	**FC**	**FT**
Asp	1.65 ± 0.05	1.65 ± 0.11	1.52 ± 0.08	1.55 ± 0.15	1.47 ± 0.28	1.49 ± 0.13	2.25 ± 0.07	2.25 ± 0.10
Thr	0.73 ± 0.02	0.72 ± 0.06	0.72 ± 0.02	0.67 ± 0.03	0.91 ± 0.15	1.00 ± 0.07	1.38 ± 0.07	1.44 ± 0.02
Ser	0.61 ± 0.01	0.66 ± 0.01^*^	0.65 ± 0.02	0.65 ± 0.02	0.57 ± 0.08	0.58 ± 0.02	1.42 ± 0.09	1.44 ± 0.03
Glu	2.50 ± 0.08	2.69 ± 0.08^*^	2.49 ± 0.15	2.57 ± 0.16	2.10 ± 0.20	2.10 ± 0.09	3.07 ± 0.18	3.11 ± 0.03
Gly	0.80 ± 0.04	0.80 ± 0.06	0.76 ± 0.00	0.88 ± 0.06^*^	0.42 ± 0.05	0.45 ± 0.07	1.22 ± 0.05	1.2 ± 0.06
Ala	1.03 ± 0.00	1.08 ± 0.02^*^	1.00 ± 0.05	1.00 ± 0.06	0.65 ± 0.13	0.70 ± 0.04	1.32 ± 0.07	1.34 ± 0.06
Cys	0.10 ± 0.01	0.09 ± 0.03	0.10 ± 0.02	0.10 ± 0.02	0.04 ± 0.02	0.09 ± 0.01^*^	0.16 ± 0.03	0.17 ± 0.06
Val	0.72 ± 0.01	0.70 ± 0.01	0.72 ± 0.04	0.71 ± 0.03	0.62 ± 0.04	0.65 ± 0.06	1.59 ± 0.09	1.59 ± 0.04
Met	0.17 ± 0.08	0.2 ± 0.02	0.21 ± 0.08	0.20 ± 0.05	0.02 ± 0.01	0.07 ± 0.05	0.38 ± 0.17	0.38 ± 0.09
He	0.68 ± 0.01	0.65 ± 0.03	0.67 ± 0.05	0.64 ± 0.04	0.48 ± 0.04	0.49 ± 0.07	1.2 ± 0.08	1.19 ± 0.03
Leu	1.16 ± 0.01	1.06 ± 0.05^*^	1.13 ± 0.09	1.07 ± 0.06	0.76 ± 0.06	0.75 ± 0.11	1.94 ± 0.11	1.94 ± 0.04
Tyr	0.62 ± 0.02	0.63 ± 0.06	0.62 ± 0.06	0.61 ± 0.03	0.49 ± 0.08	0.43 ± 0.13	1.11 ± 0.07	1.13 ± 0.04
Phe	0.74 ± 0.02	0.67 ± 0.04	0.75 ± 0.05	0.70 ± 0.06	0.58 ± 0.05	0.48 ± 0.12	1.31 ± 0.06	1.31 ± 0.02
Lys	1.34 ± 0.04	1.30 ± 0.07	1.3 ± 0.12	1.28 ± 0.08	0.84 ± 0.05	0.91 ± 0.13	1.79 ± 0.13	1.8 ± 0.06
His	0.50 ± 0.03	0.45 ± 0.03	0.46 ± 0.03	0.46 ± 0.01	0.54 ± 0.06	0.62 ± 0.02	0.96 ± 0.02	1.00 ± 0.06
Arg	1.47 ± 0.02	1.34 ± 0.14	1.55 ± 0.16	1.33 ± 0.05	0.67 ± 0.04	0.71 ± 0.15	1.71 ± 0.10	1.69 ± 0.05
Pro	0.80 ± 0.03	0.76 ± 0.06	0.71 ± 0.05	0.71 ± 0.06	1.12 ± 0.24	1.23 ± 0.14	1.16 ± 0.04	1.13 ± 0.02
EAA	5.55 ± 0.11	5.56 ± 0.36	5.77 ± 0.54	5.61 ± 0.25	4.61 ± 0.6	4.73 ± 0.22	10.34 ± 0.61	10.34 ± 0.70
T	15.63 ± 0.21	15.43 ± 0.40	15.37 ± 0.90	15.14 ± 0.64	12.29 ± 1.33	12.77 ± 0.97	23.96 ± 1.38	24.08 ± 0.64

Data were expressed as mean ± S.E.M. Different superscript letters indicate significant differences (*P* < 0.05). Only the major amino acids are shown in the table, and the detected amino acids include Asp (Aspartic acid), Thr (Threonine), Ser (Serine), Glu (Glutamic acid), Gly (Glycine), Ala (Alanine), Cys (Cysteine), Val (Valine), Met (Methionine), Ile (Isoleucine), Leu (Leucine), Tyr (Tyrosine), Phe (Phenylalanine), Lys (Lysine), His (Histidine), Arg (Arginine) and Pro (Proline). EAA, essential amino acids; TAA, total amino acids.

In the muscle tissue of both male and female crabs, the amino acids present in relatively high concentrations include Glu, Asp, Ly, and Arg, while Met exhibits the lowest concentration. When compared to the control group, dietary coconut meat supplementation could significantly increase the levels of Ser, Glu, and Ala in the muscle tissue of male crabs, while concurrently reducing the content of Leu (*P* < 0.05). In the gonadal tissue, the addition of coconut meat to the feed leads to a notable increase in Cys levels within the testes of male crabs (*P* < 0.05); however, there is no significant effect on the concentrations of other amino acids (*P* > 0.05).

### 3.5 DEGs in gonad and muscle tissues of male and female crabs

A total of 431474860 clean data were obtained after filtering, with Q20 and Q30 values higher than 97.72% and 94.41%, respectively (Table 5). The GC content ranged from 44.91% to 46.62%. These results suggested that the transcriptome data obtained from the carb were reliable.

**Table 5:** Summary of RNA-seq data.

**Sample**	**Clean data**	**Q20 (%)**	**Q30 (%)**	**GC content (%)**
MT1	46069402	97.97%	94.41%	44.93%
MT2	40792452	98.26%	95.23%	45.02%
MT3	45367002	97.72%	94.94%	45.06%
MC1	39851922	98.13%	94.97%	44.91%
MC2	38391186	98.21%	95.24%	45.10%
MC3	48454396	98.24%	95.31%	45.14%
FT1	41152034	98.42%	95.40%	46.62%
FT2	36383766	98.37%	95.34%	46.09%
FT3	38421718	98.44%	95.54%	46.02%
FC1	40885432	98.43%	95.52%	45.61%
FC2	40800860	98.44%	95.62%	45.65%
FC3	38790576	98.43%	95.61%	45.99%

MT1-MT3: the muscle tissues of the male crabs in the treatment group; MC1-MC3: the muscle tissues of the male crabs in the control group; FT1-FT3: the muscle tissues of the female crabs in the treatment group; FC1-FC3: the muscle tissues of the female crabs in the control group.

**Figure 2:**
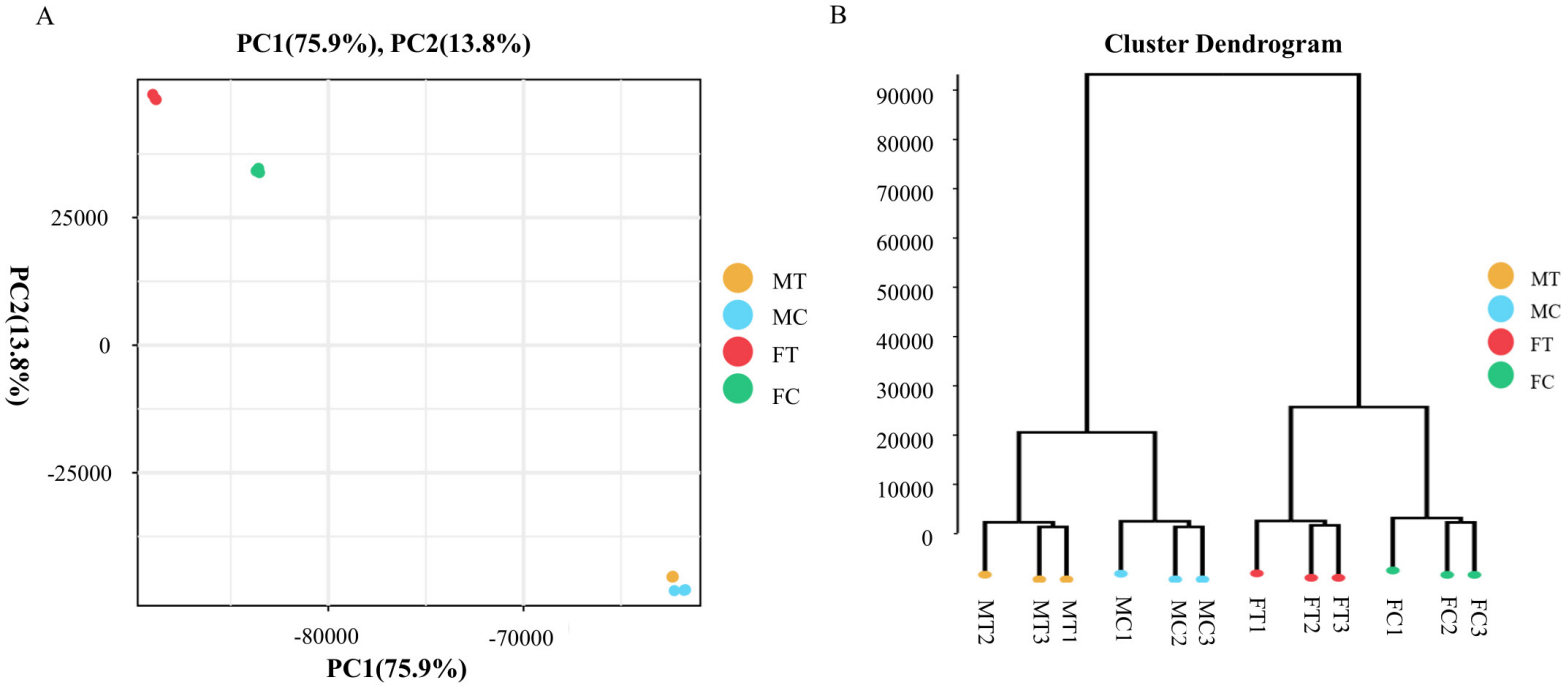
Sample relationship analysis in muscle tissue of Chinese mitten crab among control and experimental groups. **(A)** PCA analysis; **(B)** Cluster dendrogram analysis.

**Figure 3:**
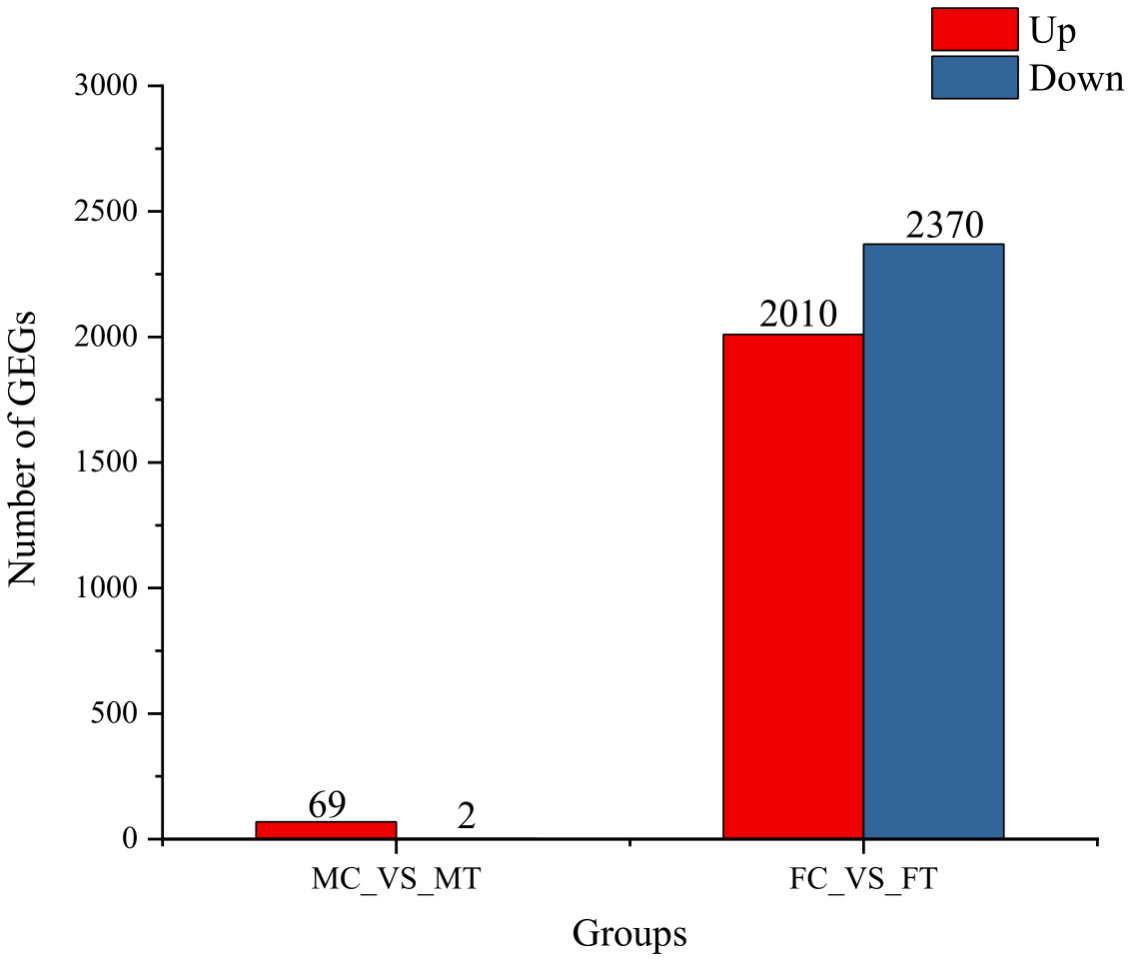
Number of significantly up-regulated and down-regulated genes in muscle of Chinese mitten crab among control and experimental groups.

The results from PCA and cluster dendrogram analyses revealed a clear separation between the samples from the control and experimental groups (Figures 2A, B). These findings indicate that the gene expression patterns in muscle tissues of crabs have undergone significant alterations following dietary supplementation with coconut meat. Specifically, a total of 71 differentially expressed genes (DEGs) were identified in the muscle tissue of male crabs, while 4,380 DEGs were detected in females. In male muscle tissue, there were 69 upregulated genes and 2 downregulated genes, whereas in female muscle tissue, 2,010 genes were upregulated and 2,370 were downregulated (Figure 3).

### 3.6 GO enrichment analysis

**Figure 4:**
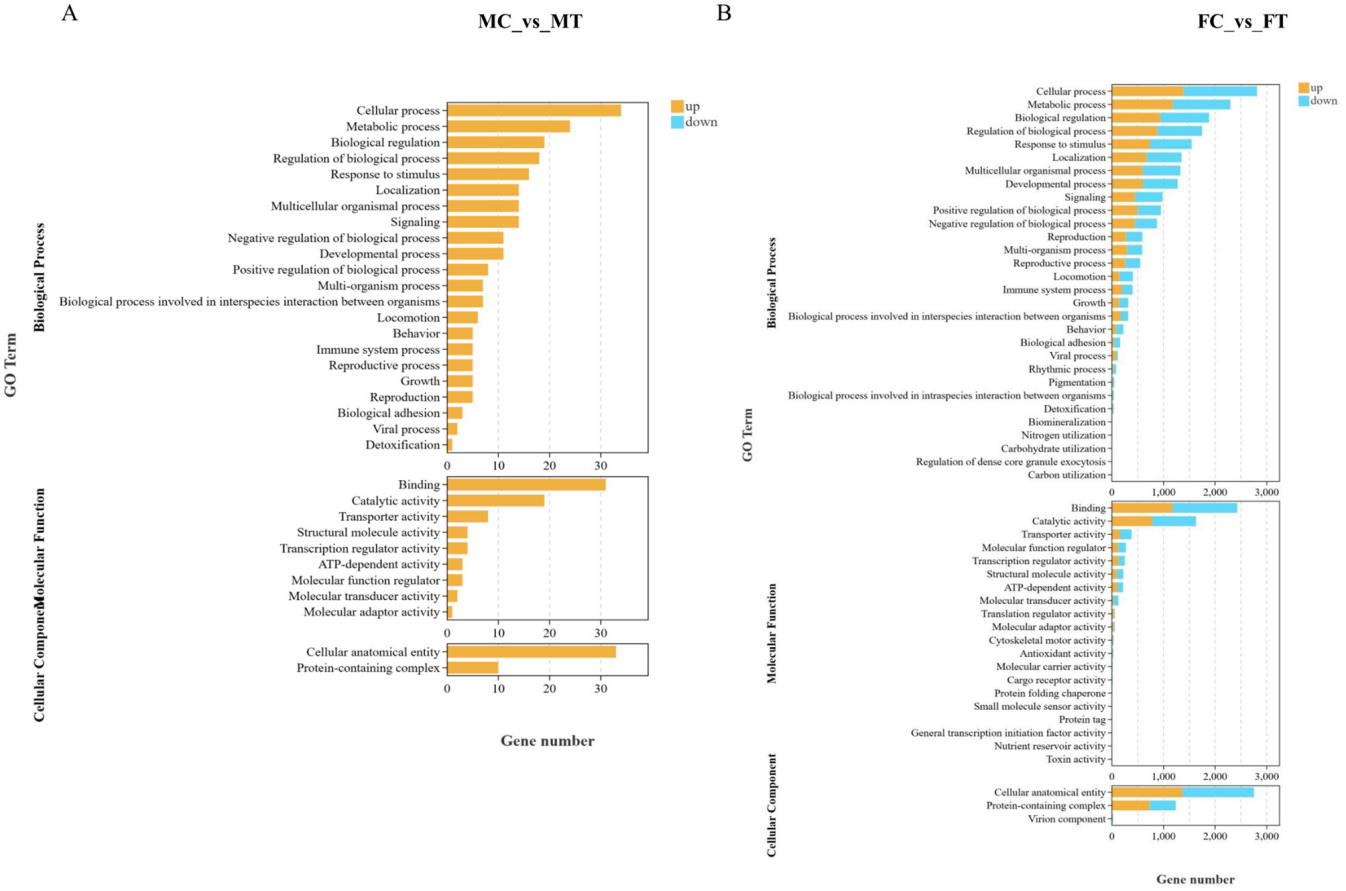
GO enrichment analysis for the DEGs in muscle of Chinese mitten crab among control and experimental groups. **(A)** DEGs enrichment in the GO for MC vs. MT; **(B)** DEGs enrichment in the GO for FC vs. FT.

Enrichment analysis was conducted to investigate the biological response to following dietary supplementation with coconut meat in the muscle tissue, specifically focusing on molecular function, cellular components, and biological processes (Figure 4). After the crabs consumed the feed that was supplemented with coconut meat, whether it is a male or a female, the DEGs were all primarily enriched in the biological process category, including cellular processes, metabolic processes, biological regulation, and regulation of biological processes. In terms of molecular function, the top two GO terms (level 2) were binding and catalytic activity. In the cellular component category, the DEGs were mainly associated with three GO terms (level 2): cell anatomical entity, and protein-containing complex.

### 3.7 KEGG enrichment analysis

**Figure 5:**
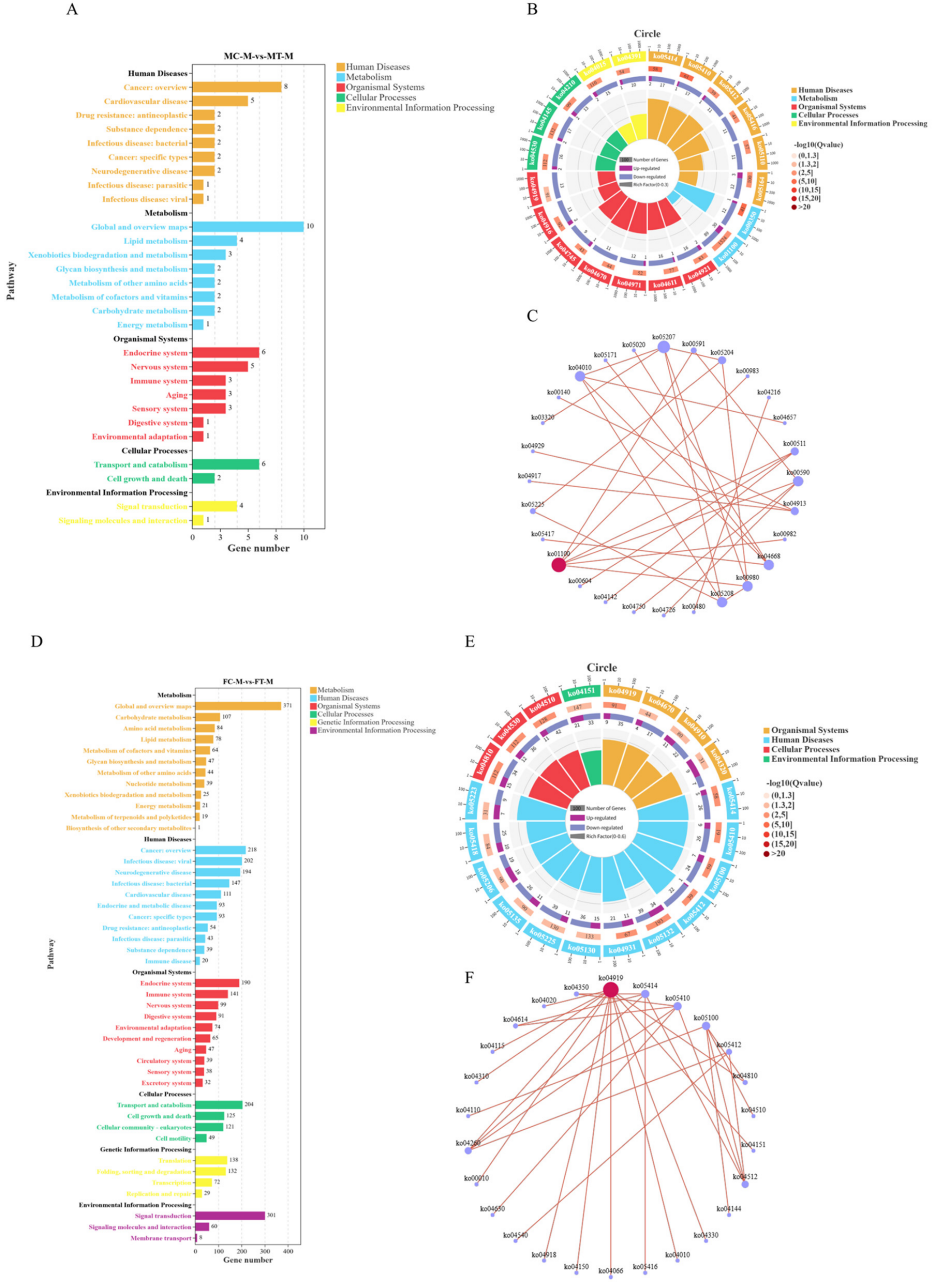
KEGG enrichment analysis for the DEGs in muscle of *E. sinensis* among control and experimental groups. **(A)** DEGs enrichment in the KEGG A Class and B Class for MC vs. MT. **(B)** The significantly enriched KEGG pathways for MC vs. MT. **(C)** Network plot of the interactions among different pathways for MC vs. MT. **(D)** DEGs enrichment in the KEGG A Class and B Class for FC vs. FT. **(E)** The significantly enriched KEGG pathways for FC vs. FT. **(F)** Network plot of the interactions among different pathways for FC vs. FT.

As illustrated in Figure 5A, a total of 23 differentially expressed genes (DEGs) were identified in male crabs, which were enriched in five KEGG Class A categories and 28 KEGG Class B categories. Dietary supplementation with coconut meat induced significant alterations in 22 pathways, and the top five signal pathways including the lipid metabolism (*P* = 0.000405), Xenobiotics biodegradation and metabolism (*P* = 0.003823), Cell growth and death (*P* = 0.006712), Immune system (*P* = 0.008983), and Glycan biosynthesis and metabolism (*P* = 0.009975) (Figure 5B, Supplementary Table S1). Notably, the network plot indicated that the Metabolic pathways (ko00110) was the primary pathway among those affected (Figure 5C).

In female crabs, a total of 1,429 DEGs were enriched in six KEGG Class A categories and 44 KEGG Class B categories (Figure 5D). Similar to males, dietary supplementation with coconut meat led to significant changes in 26 pathways, and the top five signal pathways including Signal transduction (*P* = 0.001377), Cell growth and death (*P* = 0.0034), Infectious disease: bacterial (*P* = 0.003677), Metabolism of other amino acids (*P* = 0.004258), and Signaling molecules and interaction (*P* = 0.004598) (Figure 5E, Supplementary Table S2). The network plot revealed that Thyroid hormone signaling pathway (ko04919) was the key pathway among the altered pathways (Figure 5F).

### 3.8 Candidates of internal regulation genes

KEGG analysis revealed that signaling pathways related to metabolism and Endocrine system were the main enriched pathways based on MC vs. MT, and FC vs. FT. Among these pathways, the carbohydrate metabolism, amino acid metabolism, lipid metabolism, and endocrine system were enriched in all comparisons (Figure 5). These signaling pathways may be the key intrinsic factors that cause differences in the growth and development of crabs when they are fed dietary supplementation with coconut meat compared to those fed diets without coconut meat. 39 DEGs were screened out from these pathways, which were significantly differential expression in experimental groups (Table 6).

**Table 6:** The genes with a few significantly changed KEGG pathways after fed with coconut meat.

**Signal pathway**	**DEGs**	**Gene annotation**
Carbohydrate metabolism	*Mtm1*	Myotubularin
	*PIP4K2*	1-phosphatidylinositol-5-phosphate 4-kinase
	*PTEN*	phosphatidylinositol-3,4,5-trisphosphate 3-phosphatase and dual-specificity protein phosphatase PTEN
	*TCHH*	Trichohyalin
	*IDH3*	Isocitrate dehydrogenase (NAD+)
	*ACO*	Aconitate hydratase
	*DLD*	Dihydrolipoyl dehydrogenase
Cell growth and death	*GSK3B*	Glycogen synthase kinase 3 beta
	*CCND1*	G1/S-specific cyclin-D1
	*PCNA*	Proliferating cell nuclear antigen
	*APC1*	Anaphase-promoting complex subunit 1
	*EP300*	E1A/CREB-binding protein
	*ESP1*	Separase
	*ATM*	Serine-protein kinase ATM
Amino acid metabolism	*Hpd*	4-Hydroxyphenylpyruvate dioxygenase
	*PNP*	Purine Nucleoside Phosphorylase
	*DAO*	D-amino-acid oxidase
	*ALDH*	Aldehyde dehydrogenase (NAD+)
	*ACAT*	Acetyl-CoA C-acetyltransferase
	*DHKTD1*	2-oxoadipate dehydrogenase E1 component
Lipid metabolism	*SORT*	Sortilin 1
	*SGPP1*	Sphingosine-1-phosphate phosphatase 1
	*SPT*	Serine palmitoyltransferase
	*GAL3ST1*	Galactosylceramide sulfotransferase
	*sph*	Sphingomyelin phosphodiesterase
	*SPLA2*	Secretory phospholipase A2
	*agpS*	Alkyldihydroxyacetonephosphate synthase
	*GNPAT*	Glyceronephosphate O-acyltransferase
	*LCLAT1*	Lysocardiolipin and lysophospholipid acyltransferase
	*AGPAT3_4*	Lysophosphatidic acid acyltransferase/lysophosphatidylinositol acyltransferase
Endocrine system	*Papss1*	3'-Phosphoadenosine5′-phosphosulfate synthase 1
	*PLCB*	Phosphatidylinositol phospholipase C, beta
	*MAPK1_3*	Mitogen-activated protein kinase 1/3
	*ATP1A*	Sodium/potassium-transporting ATPase subunit alpha
	*MTOR*	Serine/threonine-protein kinase mTOR
	*PIK3CA_B_D*	Phosphatidylinositol-4,5-bisphosphate 3-kinase catalytic subunit alpha/beta/delta
	*Klkb1*	Kallikrein B1
	*Egfr*	Epidermal Growth Factor Receptor
	*TLR2*	Toll-like receptor 2

### 3.9 qPCR Validation

**Figure 6:**
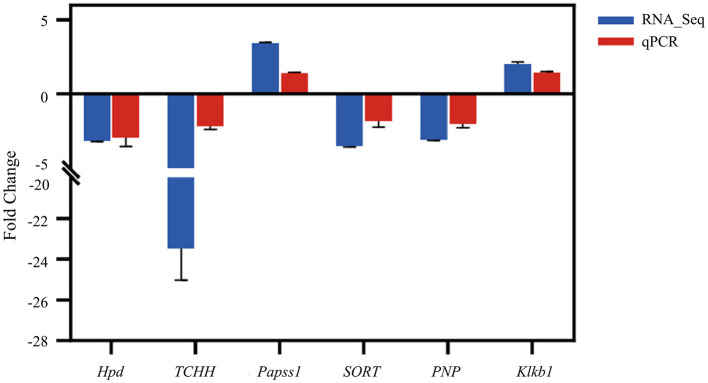
Result comparison of the qPCR and RNA-seq. The qPCR and RNA-seq results are shown in red and blue, respectively. The abscissa presents the name of DEGs; the ordinate shows the expression level.

To validate the reliability of the expression of DEGs identified via RNA-seq, six genes were randomly selected for validation using qPCR (Figure 6). The qPCR results exhibited consistent expression patterns with those obtained from RNA-seq analysis. This confirmed the accuracy and reliability of the RNA-seq experiments.

## 4 Discussion

Chinese mitten crab is an omnivorous species primarily consuming plant-based food in its natural habitat (22), demonstrating its strong capacity for plant consumption. Coconut meat, known for its rich content of crude protein, crude fat, and crude fiber, has been utilized in the formulation of livestock feed. These components may enhance the digestive enzyme activity in Chinese mitten cra, thereby improving its ability to absorb proteins and other essential nutrients (23). In this study, we observed that the average CF of female Chinese mitten crab in the experimental group was significantly higher than that of the control group (*P* < 0.05). This finding suggests that incorporating coconut meat into the feed promotes growth and development in Chinese mitten crab, aligning with the results reported by Zhang et al. (24) in poultry studies. However, our results indicated no significant difference in the GSI between the experimental and control groups. This suggests that the addition of coconut meat does not notably affect the edibility of the gonads in Chinese mitten crab.

The antioxidant capacity of serum in aquatic animals plays a crucial role in the physiological health of various tissues (25, 26). Research indicates that different feeding patterns can effectively enhance the antioxidant capacity in species such as *Cherax destructor* (27) and *Scylla paramamosain* (28). This suggests that crustaceans are highly sensitive to their dietary requirements, with feeding conditions significantly impacting the functionality of their antioxidant systems. Coconut meat has been shown to enhance both antioxidant capacity and immune function in animals (29). Moreover, Vigila et al. (30) explored the immunomodulatory effects of coconut protein in immunosuppressed Swiss albino mice, finding substantial immunomodulatory activity attributed to coconut protein. In the current study, the activities of SOD, CAT and GPx in Chinese mitten crab fed with coconut meat were significantly higher than those in the control group. T-SOD, CAT, and GPx, as the primary endogenous antioxidant enzymes, collectively protect the body from oxidative stress damage and maintain the balance between oxidation and antioxidation (31). SOD reduces oxidative damage by eliminating excess free radicals (32), while CAT protects cells from hydrogen peroxide-induced damage (33). GPx plays a complementary and crucial role by catalyzing the reduction of various lipid hydroperoxides and hydrogen peroxide, utilizing glutathione as a substrate, thereby preventing lipid peroxidation and protecting cellular membranes (34). The coordinated action of these enzymes forms a robust antioxidant defense network. The interaction and synergistic effects among SOD, CAT, and GPx are essential for efficiently neutralizing reactive oxygen species and minimizing oxidative damage (32). Higher activities of SOD, CAT, and GPx correlate with reduced cellular damage and improved overall health. T-AOC, a comprehensive indicator, evaluates the organism's antioxidant status, reflecting the protective effects conferred by the synergy of enzymatic and non-enzymatic defense systems in the Chinese mitten crab. These findings indicate that dietary supplementation with coconut meat significantly improves the antioxidant performance of this species.

The fatty acid and amino acid composition in Chinese mitten crabs can be effectively regulated through nutrient intake, which presents a viable strategy for breeding high-quality crabs (35). Flavor development in Chinese mitten crabs is closely linked to the levels of free amino acids and fatty acids. Free amino acids significantly influence the primary taste and fundamental components of aquatic organisms. Key amino acids include aspartic acid, glutamic acid, glycine, and alanine; their concentrations are vital for the meat flavor of aquatic species (36). Analysis of amino acid content in muscle revealed that the flavor amino acids in males from the experimental group exceeded those in the control group. In particular, contents of glutamic acid, glycine, and alanine in male crabs were significantly elevated compared to the control. Similarly, the levels of these four amino acids in the gonad tissues of both female and male crabs in the experimental group were higher than in the control group, though not to a statistically significant degree. These findings suggest that adding coconut meat enhances the flavor profiles of the gonad and muscle tissues in Chinese mitten crabs, with a more pronounced effect observed in muscle tissue.

Specific amino acids, such as cysteine (37), glycine (38), and glutamic acid (39), are known to support immune function, protect against infections and diseases, and mitigate excessive immune responses like autoimmunity. These amino acids were found to be significantly higher in the experimental group compared to the control group under conditions MT vs. MC for gonad tissue and FT vs. FC for muscle tissue. This study demonstrates that incorporating coconut meat into the feed can enhance the immunity of Chinese mitten crab by increasing the levels of immune-related amino acids.

In crustaceans, the nutritional regulation of fatty acid profiles has been extensively studied. For instance, dietary lipid sources significantly influence the PUFA composition in shrimp hepatopancreas and muscle, subsequently affecting growth and stress resistance (40). Similarly, in crabs, dietary n-3 PUFAs have been shown to be critical for ovarian maturation and offspring quality (41). This study found that dietary coconut meat supplementation influenced the fatty acid profiles in the muscle and gonads of Chinese mitten crab, though the differences were not statistically significant. Notably, the lauric acid (C12:0) content in the gonads of crabs fed with coconut meat was significantly higher than in those without dietary coconut meat. Lauric acid, which constitutes over 50% of coconut oil, serves as an effective food emulsifier. It not only provides rapid energy but also helps reduce fat deposition, improves insulin sensitivity, and exhibits broad-spectrum antimicrobial activity against various pathogens (42, 43). Fatty acids play a crucial role in food flavor development, primarily as key substrates in lipid oxidation (44). Flavor attributes are predominantly influenced by saturated fatty acids (SFAs) and monounsaturated fatty acids (MUFAs), while polyunsaturated fatty acids (PUFAs) primarily affect nutritional value (45). In crustaceans, PUFAs have been shown to influence growth performance (46), ovarian development (47), and reproductive performance (41). In this study, the PUFA content in the muscle and gonads of both male and female crabs was higher in the experimental group than in the control group, with significant increases observed in the gonads of female crabs and the muscle of male crabs. PUFAs also play a role in antioxidant defense, with their effects depending on content, proportion, and specific types (48). This perspective is further supported by research in *Litopenaeus vannamei*, where dietary polyunsaturated fatty acids have been demonstrated to modulate antioxidant enzyme activities and enhance immune response (49). This suggests a close relationship between PUFAs and antioxidant capacity, likely mediated through their influence on antioxidant enzyme activity and immune function. Taken together, our results on coconut meat-induced fatty acid changes are consistent with the broader understanding that dietary intervention is a potent tool for modulating the lipid nutrition and associated physiological functions in cultured crustaceans.

The development of Chinese mitten crab is closely associated with organismal metabolism, and our transcriptomic analysis revealed a more pronounced response in females compared to males. This sexually dimorphic effect is biologically plausible. Female crustaceans typically invest substantial resources into reproduction, which involves extensive vitellogenesis and lipid deposition in the gonads (50). Dietary interventions that alter nutrient availability or metabolic flux are therefore likely to trigger a stronger and broader transcriptional reprogramming in females to support these energetically costly processes (51). In both male and female crab muscle and gonad tissues, we observed significant enrichment of metabolic pathways through GO and KEGG analyses. Compared with the control group, the experimental group showed numerous differentially expressed genes primarily enriched in Cell growth and death and metabolic pathway. The results demonstrate that dietary supplementation with coconut meat significantly upregulates differentially expressed genes within the cell cycle pathway in crab muscle tissue, including *GSK3B, CCND1*, and *APC1*. *GSK3B* is a key regulator of innate immune and inflammatory responses in aquatic species, known to enhance the production of pro-inflammatory cytokines including TNF-α, IL-6, and IL-1β (52). The APC protein functions as a critical scaffolding component that assembles *GSK3B*, β-catenin, *Axin*, and other regulatory proteins into a complex, markedly increasing the phosphorylation efficiency of β-catenin by *GSK3B* (53). This mechanism maintains low intracellular levels of β-catenin and helps prevent aberrant proliferation (54). In contrast, *CCND1*—a member of the cyclin protein family—forms active complexes with *CDK4* or *CDK6* and acts as a central driver of cell cycle progression (55). In this study, the concurrent upregulation of *GSK3B, APC1*, and *CCND1* following dietary coconut meal supplementation suggests that *CCND1* overexpression enhances muscle cell proliferation activity, potentially accelerating muscle development or increasing germ cell number in crabs. This transcriptional shift provides a mechanistic basis for the observed improvement in growth performance (e.g., body weight, condition factor) by potentially accelerating muscle hypertrophy or hyperplasia. Notably, the co-upregulation of *GSK3B* and *APC1* may indicate a compensatory feedback mechanism. Intense activation of cell proliferation mediated by *CCND1* may trigger a counter-regulatory response through elevated expression of these negative regulators (*GSK3B* and *APC1*), thereby modulating proliferative signaling and maintaining tissue homeostasis.

Transcriptomic analysis revealed that dietary supplementation with coconut meat activated the arachidonic acid (ARA) signaling pathway. The upregulation of ARA metabolism genes is directly relevant to the observed phenotypic changes, and is a precursor for eicosanoids that regulate inflammation and immunity (56), linking this pathway to the enhanced antioxidant and immune capacities observed in serum enzyme assays. Furthermore, ARA is a critical component of phospholipid membranes and is selectively retained in gonadal tissues (57, 58). Its metabolic activation likely supports the altered fatty acid profiles found in the nutrient composition analysis. This aligns with findings that wild fish with higher ARA content exhibit superior reproductive performance (59).

In the muscle transcriptome analysis, we observed a significant upregulation of *Egfr* expression in the experimental group (FDR < 0.05). This finding bridges the transcriptional response to the physiological outcomes in two key ways. First, EGFR signaling is a master regulator of tissue growth and development (60). Its activation provides a direct molecular explanation for the promoted muscle development inferred from the significantly higher condition factors in the treatment group (61). Additionally, EGFR interacts with key immune pathways (62). In our analysis, DEGs were significantly enriched in the JAK-STAT and MAPK pathways, which are pivotal for immunomodulation. Mechanistically, *Egfr* regulates cellular proliferation and immune responses through activation of MAPK-ERK, PI3K-Akt-mTOR and other signaling pathways (63, 64). Therefore, the upregulation of *Egfr* in crabs fed coconut meat likely orchestrates a coordinated response that not only enhances growth but also bolsters immune competence, thereby connecting the transcriptomic change to the improved antioxidant and health status observed at the organismal level.

## 5 Conclusion

In summary, this study evaluated the effects of dietary coconut meal supplementation on nutritional quality and molecular mechanisms in Chinese mitten crab. The addition of coconut meal significantly improved the nutritional value of gonads and enhanced the flavor-associated compounds in muscle tissue, as evidenced by optimized fatty acid and amino acid profiles. Furthermore, elevated antioxidant enzyme activities indicated a boost in overall antioxidant capacity. At the molecular level, transcriptomic analysis revealed that coconut meal influenced muscle development and metabolic regulation through key pathways including carbohydrate metabolism, lipid metabolism, amino acid metabolism, cell growth and death, and endocrine system. A total of 39 critical genes—such as *Egfr, GSK3B*, and *Mtm1*—were identified to be involved in these processes, providing mechanistic insight into the observed physiological improvements. These findings demonstrate the potential of coconut meal as a functional feed ingredient to enhance nutritional quality and modulate molecular pathways related to growth and metabolism in Chinese mitten crab aquaculture.

## Data Availability

The data presented in the study are deposited in the NCBI Sequence Read Archive (SRA) repository, accession number PRJNA1178569.
