# Supplementary material for: Dietary supplementation of coconut meat modulates growth performance, nutritional composition, and internal regulation in Chinese mitten crab (Eriocheir sinensis)
Source: Front Nutr. 2026 Mar 6;13:1757972. doi: 10.3389/fnut.2026.1757972 (PMC13002806; doi:10.3389/fnut.2026.1757972)
Supplement: Supplementary file 1 [file Table_1.docx]

Table S1. Coconut Meat Supplementation Significantly Alters KEGG Class B Pathways in Male Crabs

| Number | KEGG Class B categories | *P*value |
| --- | --- | --- |
| 1 | lipid metabolism | 0.000405 |
| 2 | Xenobiotics biodegradation and metabolism | 0.003823 |
| 3 | Cell growth and death | 0.006712 |
| 4 | Immune system | 0.008983 |
| 5 | Glycan biosynthesis and metabolism | 0.009975 |
| 6 | Chemical carcinogenesis- receptor activation | 0.015582 |
| 7 | Endocrine system | 0.016245 |
| 8 | Arachidonic acid metabolism | 0.016245 |
| 9 | Signal transduction | 0.016881 |
| 10 | Drug metabolism - cytochrome P450 | 0.016881 |
| 11 | Metabolism of xenobiotics by cytochrome P450 | 0.019529 |
| 12 | Drug resistance: antineoplastic | 0.023806 |
| 13 | Chemical carcinogenesis - reactive oxygen species | 0.023806 |
| 14 | Metabolism of other amino acids | 0.024553 |
| 15 | Aging | 0.028433 |
| 16 | Nervous system | 0.030872 |
| 17 | Sensory system | 0.038666 |
| 18 | Transport and catabolism | 0.03876 |
| 19 | Substance dependence | 0.040491 |
| 20 | Infectious disease: bacterial | 0.042348 |
| 21 | Cardiovascular disease | 0.044238 |
| 22 | Chemical carcinogenesis - DNA adducts | 0.04959 |
