# Supplementary material for: Dietary supplementation of coconut meat modulates growth performance, nutritional composition, and internal regulation in Chinese mitten crab (Eriocheir sinensis)
Source: Front Nutr. 2026 Mar 6;13:1757972. doi: 10.3389/fnut.2026.1757972 (PMC13002806; doi:10.3389/fnut.2026.1757972)
Supplement: Supplementary file 2 [file Table_2.docx]

Table S2. Coconut Meat Supplementation Significantly Alters KEGG Class B Pathways in Female Crabs

| Number | KEGG Class B categories | *P*value |
| --- | --- | --- |
| 1 | Signal transduction | 0.001377 |
| 2 | Cell growth and death | 0.0034 |
| 3 | Infectious disease: bacterial | 0.003677 |
| 4 | Metabolism of other amino acids | 0.004258 |
| 5 | Signaling molecules and interaction | 0.004598 |
| 6 | Transport and catabolism | 0.004582 |
| 7 | Dilated cardiomyopathy | 0.00459 |
| 8 | Cardiovascular disease | 0.004881 |
| 9 | Bacterial invasion of epithelial cells | 0.004806 |
| 10 | Cardiovascular disease | 0.00536 |
| 11 | Cell motility | 0.00549 |
| 12 | Tight junction | 0.00113 |
| 13 | Cellular community - eukaryotes | 0.001216 |
| 14 | Salmonella infection | 0.001451 |
| 15 | Endocrine and metabolic disease | 0.001451 |
| 16 | Infectious disease: bacterial | 0.011912 |
| 17 | Cancer: specific types | 0.011912 |
| 18 | Shigellosis | 0.012563 |
| 19 | MicroRNAs in cancer | 0.012563 |
| 20 | Cardiovascular disease | 0.012563 |
| 21 | Immune system | 0.018415 |
| 22 | Endocrine system | 0.018415 |
| 23 | Development and regeneration | 0.021699 |
| 24 | Cancer: specific types | 0.021699 |
| 25 | Endometrial cancer | 0.030407 |
| 26 | Infectious disease: viral | 0.049433 |
